# Thermal transport across wrinkles in few-layer graphene stacks†

**DOI:** 10.1039/d0na00944j

**Published:** 2021-01-13

**Authors:** A. Mohapatra, S. Das, K. Majumdar, M. S. Ramachandra Rao, Manu Jaiswal

**Affiliations:** Graphene and 2D Systems Laboratory, Department of Physics, Indian Institute of Technology Madras Chennai 600036 India manu.jaiswal@iitm.ac.in +91-44-2257-4893; Nano Functional Materials Technology Centre and Materials Science Research Centre, Department of Physics, Indian Institute of Technology Madras Chennai 600036 India msrrao@iitm.ac.in +91-44-2257-4872; Department of Electrical Communication Engineering, Indian Institute of Science Bangalore 560012 India

## Abstract

Wrinkles significantly influence the physical properties of layered 2D materials, including graphene. In this work, we examined thermal transport across wrinkles in vertical assemblies of few-layer graphene crystallites using the Raman optothermal technique supported by finite-element analysis simulations. A high density of randomly oriented uniaxial wrinkles were frequently observed in the few-layer graphene stacks which were grown by chemical vapor deposition and transferred on Si/SiO_2_ substrates. The thermal conductivity of unwrinkled regions was measured to be, *κ* ∼ 165 W m^−1^ K^−1^. Measurements at the wrinkle sites revealed local enhancement of thermal conductivity, with *κ* ∼ 225 W m^−1^ K^−1^. Furthermore, the total interface conductance of wrinkled regions decreased by more than an order of magnitude compared to that of the unwrinkled regions. The physical origin of these observations is discussed based on wrinkle mediated decoupling of the stacked crystallites and partial suspension of the film. Wrinkles are ubiquitous in layered 2D materials, and our work demonstrates their strong influence on thermal transport.

## Abstract 2

Thermal transport is strongly influenced by wrinkles in films of few-layer graphene crystallites with rotational stacking faults, as revealed from Raman optothermal measurements.

## Graphical abstract

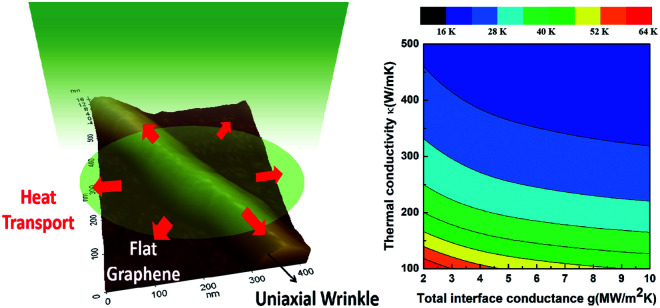

## 1 Introduction

Graphene is an important contender for thermal management applications in electronic and opto-electronic devices. The ultrahigh thermal conductivity of graphene stems from its unique phonon structure, where low energy acoustic phonon modes play a dominant role.^1–4^ High symmetry of graphene lattice, the low mass of carbon atoms, and high force constant associated with sp^2^ covalent bonds make phonons the dominant heat carriers in this system.^5–7^ The lattice thermal conductivity of suspended single-layer graphene was obtained as ∼3000–4000 W m^−1^ K^−1^, higher than that of the best bulk thermal conductors such as graphite and diamond.^2,8–10^ The thermal conductivity of supported single- and few-layer graphene is further quenched due to the additional scattering of ZA phonon modes from the substrate.^11^

Deviations from the ideal chemical structure or morphology can have a significant bearing on heat transport. In the case of three-dimensional nanostructured systems, the porosity and architecture significantly influence the thermal properties and thermal transport.^12,13^ It has been experimentally observed that the presence of vacancies, isotopic impurities, stacking faults, and different edges reduces the thermal conductivity of graphene. In one study, *κ* of graphene was probed under low energy electron beam irradiation. It was found that *κ* decreases from 1800 W m^−1^ K^−1^ to 400 W m^−1^ K^−1^ with an increase in defect density *N*_d_ by one order of magnitude.^14^ The presence of isotope impurity ^13^C was found to adversely affect the thermal conductivity of graphene. The value of *κ* decreased from ∼2800 W m^−1^ K^−1^ for *N*_isot_ = 0.01% to *κ* ∼ 1600 W m^−1^ K^−1^ for *N*_isot_ = 50%.^15^ Li *et al.* observed a decrease in thermal conductivity of twisted bilayer graphene (T-BLG) by a factor of ∼1.4 from the value of its parent AB-stacked or Bernal BLG.^16^ Apart from defects, deviation from flat morphology associated with the local curvature in graphene also influences the thermal transport. Ripples and wrinkles in graphene are ubiquitous and are formed due to the residual stress caused by various factors such as thermal expansion mismatch with the substrate, presence of impurities, release or application of external stress, edge stresses, surface tension of transferring liquid and random thermal fluctuations. Individual wrinkles are typically uniaxial, whereas the distribution of a set of wrinkles can be either quasi-periodic or random in orientation, depending on the nature of competing effects responsible for their generation.^17^ In some cases, wrinkles can span over the entire graphene sample. These are greatly stressed along the wrinkling direction and are compressed along the texture direction.^18^ Recently, heat conduction across graphene wrinkles was studied theoretically.^18–20^ The important observations included a decrease in thermal conductivity upon an increase of the degree of wrinkling and anisotropic heat flow between texture and wrinkling directions. In these calculations, the decrease in *κ* was attributed to phonon mode localization, phonon mode softening and increased phonon scattering rate corresponding to a reduced phonon mean free path. Similarly, a more relaxed structure was observed to possess higher thermal conductivity. Experimentally, Chen *et al.* studied the thermal conductivity of wrinkled suspended single-layer graphene. Based on statistics of data from 12 samples probed with Raman mapping, the authors inferred a 27% average decrease in thermal conductivity compared to the unwrinkled case.^21^ Enhancement of chemical activity is also observed in corrugated graphene.^22^ While supported graphene can be engineered with different wrinkle geometries, the subject of phonon transport through them has not received adequate attention. Wrinkles may be found in supported graphene layers either when graphene is a sandwich thermal management layer or an active over-layer. The study of the effects of local curvature on heat flow in graphene and related systems, therefore, becomes quite important.

In this paper, we discuss thermal transport across wrinkles in vertical assemblies of few-layer graphene crystallites probed using the Raman optothermal technique, complemented with finite element analysis (FEA). Graphene samples prepared using the chemical vapor deposition (CVD) technique were found to consist of vertical assemblies of few-layer graphene crystallites with rotational stacking faults. The graphene layers were transferred onto a Si/SiO_2_ substrate and upon examination were found to possess a random network of uniaxial wrinkles with significantly variable density. Thermal transport measurements were performed using the Raman optothermal technique, both on unwrinkled and wrinkled locations. Local enhancement of thermal conductivity accompanied by a decrease in interfacial thermal conductance is observed at the wrinkle sites. The physical origin of these observations is discussed.

## 2 Experimental

Few-layer graphene samples were grown on a nickel substrate using the chemical vapor deposition (CVD) technique. The main furnace zone was kept at 850 °C and the substrate was initially annealed for 15 min at an inert Ar gas flow rate of 100 sccm. In place of conventional gaseous precursors like methane and hydrogen, camphor (C_10_H_16_O) was utilized as a green solid precursor. Detachment of the methyl groups attached to hexagonal and pentagonal carbon rings of camphor is followed by fusion of these rings to form the graphene layers on the Ni substrate. 6 mg of the solid precursor was flash heated at a temperature of 180 °C upstream of the main heating zone. At this temperature, the solid precursor sublimated in approximately 3 min and was carried into the main furnace zone with the help of Ar carrier gas, which was maintained at 100 sccm throughout the experiment. Growth was allowed to happen for 2 min followed by rapid quench cooling of the substrate using a magnetic rod arrangement. Similar growth conditions have been reported in the literature involving upstream sublimation of camphor that is carried to the main furnace zone by Ar carrier gas, which is followed by pyrolysis in the main furnace at 800 °C for 3 min and quench cooling of the Ni substrate.^23^ Note that the somewhat longer total camphor exposure times in our work resulted in higher graphene film thickness. After the growth, graphene was dry-transferred onto the Si/SiO_2_ substrate using thermal release tape for further studies. The topography of the obtained graphene samples was studied using a Park systems NX-10 atomic force microscopy setup in non-contact mode. Raman spectroscopy of the samples along with power-dependent Raman studies were carried out using a WITec alpha300 R Raman spectrometer at a laser wavelength of 532 nm (2.33 eV) along with 1800 grooves per mm grating. Variable temperature Raman scattering measurements were carried out using a Linkam THMS-600 micro-thermoelectric cell and Horiba HR-800-UV Raman spectrometer with an Argon ion laser of 488 nm (2.54 eV) excitation wavelength. The incident laser power is maintained below 250 μW to avoid temperature rise of the sample due to intrinsic contribution. The thermal transport measurements and analysis procedures were validated using mechanically exfoliated graphene control samples having comparable thickness.

## 3 Results and discussion

**Fig. 1:**
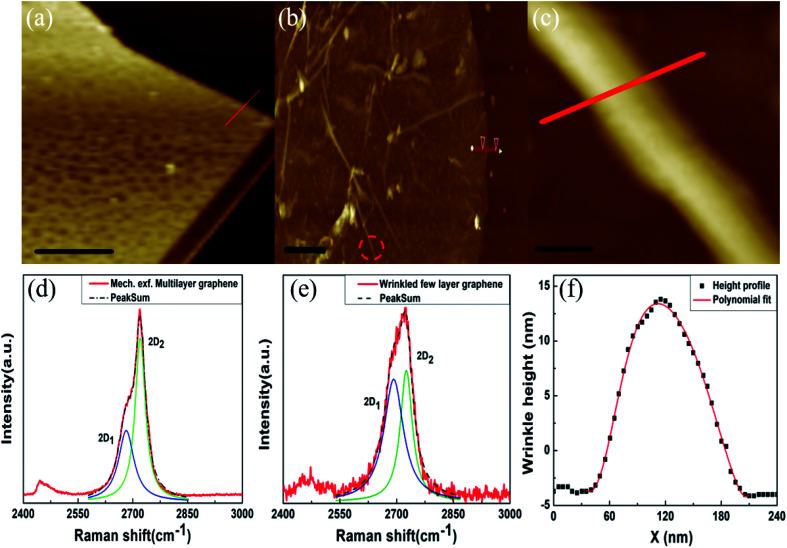
(a) AFM topography image of mechanically exfoliated multi-layer graphene (scale bar is 2.5 μm), (b) AFM topography image of wrinkled few-layer graphene (scale bar is 2 μm), (c) high-resolution AFM image of the wrinkle (scale bar is 0.1 μm), (d) Raman spectrum showing the 2D band of mechanically exfoliated multi-layer graphene with its deconvolutions, (e) Raman spectrum showing the 2D band of wrinkled few layer graphene together with its deconvolutions, (f) height profile of a graphene wrinkle along the red-line indicated in ‘part (c)’.

We first examine the morphology and thickness of these samples using AFM topography imaging and micro-Raman spectroscopy. Throughout our studies, mechanically exfoliated samples with thickness comparable to the CVD grown samples are used as control samples. The AFM topography image of mechanically exfoliated multi-layer graphene is shown in Fig. 1(a): the estimated thickness is ≈9 nm (see ESI Fig. S1†), and the morphology is characteristically flat and unwrinkled. Fig. 1(b) shows the non-contact mode AFM topography image of the CVD grown graphene sample, transferred on the Si/SiO_2_ substrate. Randomly-oriented wrinkle formations can be clearly seen in the figure. Lengths of these formations are very high, ≈4 μm, and each wrinkle is unidirectional in nature. The estimated thickness of CVD graphene from the height profile image is ≈15 nm (see Fig. S1†), which points to multilayer graphene. Note that the thickness modulation due to wrinkling is over and above this thickness value. We note that even when the thickness of the CVD graphene film is high, the wrinkle patterns observed are surprisingly similar to those reported for single- or few-layer graphene.^24^ This aspect is discussed in detail, subsequent to the presentation of Raman spectra. Micro-Raman spectroscopy measurements were carried out to obtain further details about the layer numbers associated with the given samples. Fig. 1(d) and (e) show the 2D peaks of the Raman spectrum of the mechanically exfoliated multi-layer graphene sample and wrinkled regions of the CVD grown sample, respectively. The 2D mode involves a two-phonon process of interband scattering between *K* and *K*′ valleys. Since the presence of electronic sub-bands influences the scattering processes, the line shape and width of the 2D mode are sensitive to the thickness of the graphene samples.^25^ In particular, the 2D_1_ (lower wavevector) and 2D_2_ (higher wavevector) deconvoluted peaks are of comparable intensity in few-layer graphene, while the 2D_2_ intensity becomes twice as strong as 2D_1_ in multi-layer graphene or graphite.^25^ The 2D peak for mechanically exfoliated multi-layer graphene was deconvoluted and was consistent with the fitting of 2 Lorentzian peaks, 2D_1_ and 2D_2_ as shown in Fig. 1(d). The position of 2D_1_ is ∼2681.96 cm^−1^ with the corresponding FWHM ∼51.11 cm^−1^ and the position of 2D_2_ is ∼2720.36 cm^−1^ with the corresponding FWHM ∼33.83 cm^−1^. The 2D-peak of mechanically exfoliated graphene shows a clear signature of multi-layer graphene or graphite, consistent with the literature.^25,26^ The 2D peak for wrinkled regions of CVD graphene was deconvoluted and it was consistent with the fitting of 2 Lorentzian peaks, 2D_1_ and 2D_2_ as shown in Fig. 1(e). The position of 2D_1_ is ∼2694.09 cm^−1^ with the corresponding FWHM ∼64.88 cm^−1^ and the position of 2D_2_ is ∼2726.58 cm^−1^ with the corresponding FWHM ∼41.72 cm^−1^. Raman signatures from CVD grown graphene can be ascribed to those of few-layer graphene, despite the overall film thickness, and these signatures point to the existence of stacking faults between the vertically assembled few-layer crystallites. The 2D peak profile of our wrinkled sample is consistent with that of 5-layered graphene, as studied rigorously in the literature.^16,25–28^ More details are provided in the ESI (see Fig. S2†). Similarly, observations from unwrinkled regions of CVD graphene are shown in Fig. S3.† The Raman 2D peak of unwrinkled regions of the CVD sample is consistent with the spectrum of 4-layered graphene as shown in Fig. S3.† The line-shape and deconvolution of the 2D peak clearly do not indicate the signatures of multi-layer CVD graphene for both wrinkled and unwrinkled regions. A detailed comparison of the Raman 2D peak acquired with different laser wavelengths for wrinkled and unwrinkled locations is given in Fig. S2.† In addition to the Raman signatures, the existence of a random network of uniaxial wrinkles is another piece of evidence that the multi-layer CVD graphene is composed of few-layer graphene stacks, since the large bending rigidity of the latter precludes wrinkle formation, as discussed below. Fig. 1(c) shows the high resolution AFM topography image of a representative wrinkle, measured at the location marked with a red dashed circle in Fig. 1(b). As shown in Fig. 1(f), the acquired line profile is fitted with a polynomial function, and from the fitting parameters the aspect ratio (*ζ*) is calculated to be *ζ* > 10.^17,29^ The wrinkle line profile is well described with a Gaussian curve with wavelength *λ* ∼98.44 nm and amplitude *A* ∼ 8.66 nm. Hence the wrinkling level^19^*γ* = [*A*/*λ*] × 100% is calculated to be 8.79%. While the wrinkle morphology bears a strong resemblance to similar morphologies noted in single-layer graphene,^24^ it is intriguing that these samples should show any wrinkling at all for this thickness which is 30-fold higher, given that the bending rigidity of a thin film increases as the cube of the film thickness.^30^ For comparison, mechanically exfoliated graphene samples with even lesser thickness and which lack stacking disorder and are cleaved on identical Si/SiO_2_ substrates show a complete lack of wrinkling as observed from the AFM topography image and height profile image shown in Fig. 1(a) and S1(a)† respectively. Thus, without stacking faults between the vertical assemblies of few-layer graphene crystallites, it may otherwise be impossible to generate a network of wrinkles in these thickness regimes. To summarize, AFM and Raman studies indicate that CVD grown graphene samples consist of stacks of few-layer graphene crystallites arranged vertically with rotational stacking faults, and these observations are consistent across wrinkled and unwrinkled regions (see Raman maps in the ESI Fig. S4†). Our results based on Raman spectroscopy and AFM should also be compared with the observations of Kalita *et al.*, who have demonstrated that pyrolysis of camphor results in the formation of rotational stacking faults in few-layer CVD graphene on a Ni substrate.^23^ In that work, the authors had synthesized multi-layer graphene films using growth conditions quite comparable to the one used by us, as discussed in the Experimental Section above. Their work emphasized the formation of rotational stacking faults between graphene layers as evidenced from relative rotations by variable angles of the set of hexagonal diffraction spots observed in high resolution transmission electron microscopy (HRTEM), considered together with the results from Raman spectroscopy.^23^ See ESI Fig. S5† for our TEM results showing the misorientation.

The Raman optothermal technique has been one of the most effective methods to investigate thermal transport in graphene and related systems.^1,2,8^ Towards this objective, the G peak shift was measured as a function of the incident laser power for all the sample types using two different objectives, 100× (with NA – 0.9 and spot radius *r*_0_ ∼ 0.360 μm) and 50× (with NA – 0.55 and *r*_0_ ∼ 0.590 μm). Details of the calibration procedures are presented in the ESI (see ESI Section VI).† Across the entire range of measurements, the incident power level is kept below a maximum of 7 mW. For this maximum applied power, no increase in the D peak intensity is observed. The area integrated ratio of the defect-mode peak normalized with the G-peak, *I*_D_/*I*_G_ < 0.09 for the lowest and highest laser power used; such small intensities for the defect peak are also reported for pristine graphene on Si/SiO_2_.^31^

**Fig. 2:**
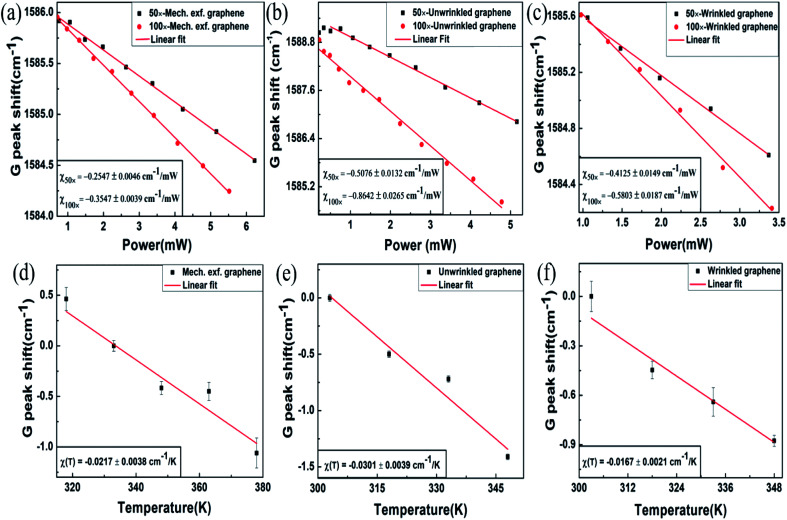
Raman G-band shift with laser power variation with two different incident energy densities, using 100× and 50× objectives for: (a) mechanically exfoliated multi-layer graphene, (b) unwrinkled sites in CVD graphene, and (c) wrinkled sites in CVD graphene; variable temperature measurements of the G peak shift for: (d) mechanically exfoliated multi-layer graphene, (e) unwrinkled sites in CVD graphene, and (f) wrinkled sites in CVD graphene.

Fig. 2(a) shows the G peak shift *versus* incident laser power at both objectives for mechanically exfoliated multi-layer graphene. The G peak shows a red shift with increase in incident laser power. This can be attributed to intrinsic contributions such as thermal expansion, anharmonicity and extrinsic contribution from thermal expansion coefficient mismatch induced strain.^32,33^ The slope of the G peak shift with incident laser power for 50× objective was found to be *χ*_50×_ = −0.2547 ± 0.0046 cm^−1^ mW^−1^, and for 100× objective *χ*_100×_ = −0.3547 ± 0.0039 cm^−1^ mW^−1^. The power dependent G-peak shift measurements for unwrinkled locations of CVD grown samples yield a slope of *χ*_50×_ = −0.5076 ± 0.0132 cm^−1^ mW^−1^ and *χ*_100×_ = −0.8642 ± 0.0265 cm^−1^ mW^−1^ for 50× and 100× objectives respectively (Fig. 2(b)). For wrinkled regions of CVD graphene the corresponding slopes are *χ*_50×_ = −0.4125 ± 0.0149 cm^−1^ mW^−1^ and *χ*_100×_ = −0.5803 ± 0.0187 cm^−1^ mW^−1^, as shown in Fig. 2(c). The G-peak shifts associated with all the samples follow the expected trend of higher temperature rise with larger incident energy density as discussed in the literature.^32,33^ The magnitude of temperature rises for each of the above experiments can be obtained by calibrating the G-peak shift with temperature at low-enough incident laser power. Variable temperature measurements were performed using a Linkam THMS-600 low temperature micro thermoelectric cell. The temperature of the stage was increased at an interval of 15 K and the G peak shift data for all the cases were recorded. The incident laser power was maintained below 250 μW in order to avoid any temperature rise due to intrinsic contribution. Fig. 2(d)–(f) shows the G peak shift with increase in temperature for mechanically exfoliated multi-layer graphene, unwrinkled sites of CVD graphene and wrinkled sites of CVD graphene, respectively. The obtained slopes for these cases are given by, *χ*(*T*) = −0.0217 ± 0.0038 cm^−1^ K^−1^ (exfoliated graphene), −0.0301 ± 0.0039 cm^−1^ K^−1^ (unwrinkled site of CVD graphene) −0.0167 ± 0.0021 cm^−1^ K^−1^ (wrinkled site of CVD graphene). Since the temperature rises for two different incident laser powers are known, the estimates of thermal conductivity can be made from the solutions of the heat diffusion equation, as described below.

The steady-state temperature distribution, *T*(*r*), in the examined film upon laser irradiation can be obtained by solving the following set of heat diffusion equations as extensively discussed in the literature:^2,34^1
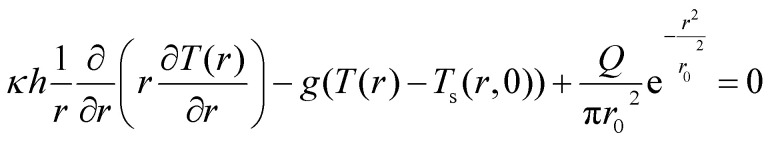
2
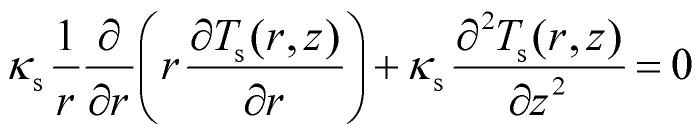


With the following boundary conditions:3
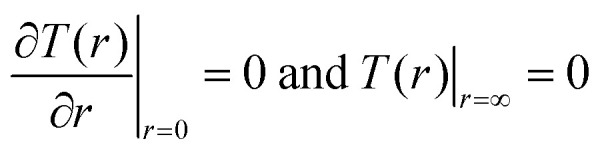
4
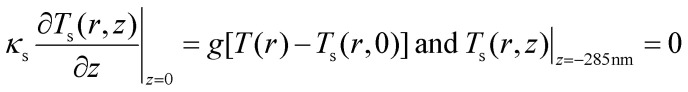
Here, *κ* is the thermal conductivity of graphene, and *h* is the corresponding film thickness. *g* is the total interface conductance and *T*_s_(*r*,0) is the temperature of the substrate at the interface. *z* = 0 is taken as the interface between the film and the substrate. The last term in the heat diffusion equation represents the source term and *r*_0_ is the incident laser spot radius which depends upon the numerical aperture (NA) of the objective. The incident laser power distribution is taken to be Gaussian, which is represented by the source term in the equation. The average temperature rise of the film upon irradiation is calculated from *T*(*r*), following the equation:5
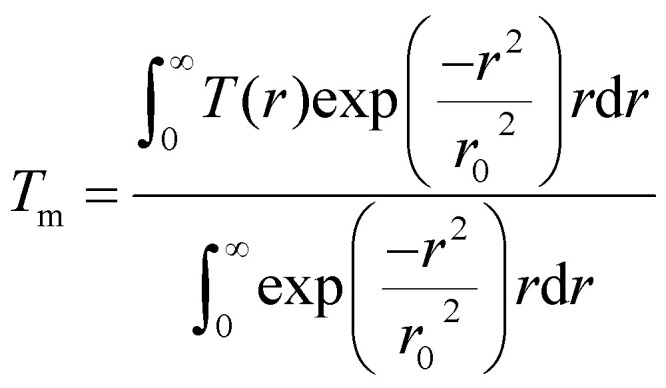


We did not observe any shift in the fundamental Raman peak of silicon at 520.7 cm^−1^ with increasing incident laser power in our experiments. Hence the temperature rise of the Si/SiO_2_ interface is considered to be negligible, even as the temperature rise in SiO_2_ was considered. As the experiments were carried out under ambient conditions and the thermal conductivity of air is very low ∼0.025 W m^−1^ K^−1^, losses due to convection are negligible and radiation contribution is also negligible.^34,35^ The calculated temperature distribution of the graphene film is a function of material and optical parameters. Equation parameters such as NA, *r*_0_, *Q* and *z* can be obtained easily as they are system/optical parameters whereas *κ* and *g* are material parameters and these need to be extracted by solving for two different temperature rises associated with the respective incident laser energy densities. In our case, we obtained two different temperature rises by utilizing objective lenses with different magnification power.^2,34^

**Fig. 3:**
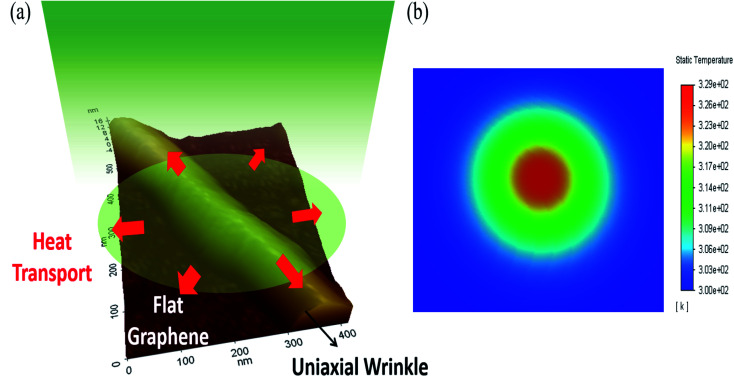
(a) Graphical representation of the heat transport in wrinkled locations overlaid on a 3D AFM topography image taken at the wrinkle site, (b) representative finite element simulation of the temperature distribution in a graphene film. The following parameters were used for calculation of the temperature distribution: film thickness ≈ 13.5 nm, *κ* = 200 W m^−1^ K^−1^ and *g* = 6 MW m^−2^ K^−1^. Incident laser power of 1 mW is considered using a 50× objective lens with NA = 0.55. The thermal conductivity of the dielectric layer is taken to be 1.37 W m^−1^ K^−1^.

We performed finite element analysis (FEA) to evaluate the thermal conductivity and total interface conductance of the films. Details of the modelling are discussed in the ESI Fig. S6.†*Q* = *nαP* represents the total power absorbed by the film where *α* is the absorption coefficient of single-layer graphene and *n* is the layer number. Here *P* is chosen as 1 mW for our calculations and total absorption is calculated based on the consideration that each layer behaves as an independent atomic sheet. Hence the total absorption is the superposition of absorption by individual layers with *α*_SLG_ ∼ 2.3%.^36^Fig. 3(a) is a schematic representation of the Raman optothermal measurements carried out on the wrinkle location, together with the radially outward heat flow in the system. While the presence of the wrinkle can introduce an angular dependence of the radially outward heat flow, a locally uniform effective *κ* and *g* are considered in our model for characterizing the heat transport in the wrinkled region, to allow for extraction of these two unknown parameters. It may be noted that this scenario is not unique to our heat transport experiment, and these considerations equally apply, for example, to thermal transport studies in suspended single-layer graphene where quasi-periodic wrinkle arrays are frequently observed due to residual strain. Fig. 3(b) shows a representative finite-element simulation result for temperature distribution in a graphene film obtained with 50× objective. The Gaussian distribution of temperature in the film upon irradiation with a laser is shown.

**Fig. 4:**
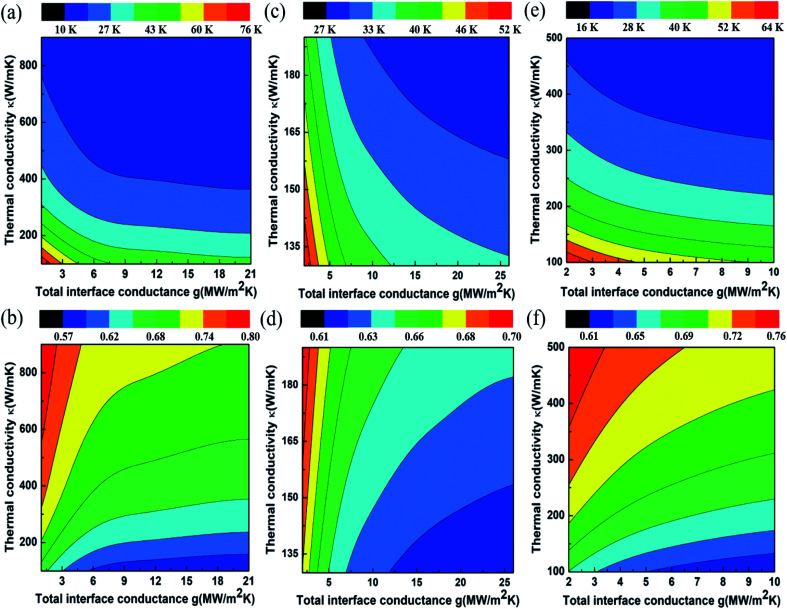
Results of FEA simulation: (a) temperature rise of mechanically exfoliated multi-layer graphene stacks with 100× objective, (b) ratio of the temperature rise *T*_50×_/*T*_100×_ for mechanically exfoliated graphene stacks, (c) temperature rise of unwrinkled few-layer graphene with 100× objective, (d) *T*_50×_/*T*_100×_ for unwrinkled few-layer graphene, (e) temperature rise of wrinkled few-layer graphene with 100× objective, (f) *T*_50×_/*T*_100×_ for wrinkled few-layer graphene.

We next discuss the estimation of the thermal conductivity, with the help of FEA simulations. Individual models are made for each sample, factoring the layer number in each case. We first validate the procedure by estimating the thermal conductivity and interface thermal conductance of mechanically exfoliated multi-layer graphene. The computed temperature rise for the exfoliated sample for 100× objective is shown in Fig. 4(a) (50× objective data are shown in ESI Fig. S7†). As can be seen from Fig. 4(a), lower total interface conductance or lower thermal conductivity is associated with higher temperature rise of the film. With an increase in *g* which corresponds to improved interface, the value of Δ*T* decreases, thereby showing the self-consistency of the calculations. The ratio of temperature rise *T*_50×_/*T*_100×_ for mechanically exfoliated graphene is shown in Fig. 4(b). In order to obtain the values of *κ* and *g* uniquely, the intersection of experimentally inferred contours is taken in the *κ*–*g* plane. One of these contours is the temperature rise obtained with 100× objective, while the other is the ratio (*T*_50×_/*T*_100×_) contour, as shown in Fig. 4(a) and (b), respectively. The thermal conductivity of mechanically exfoliated multi-layer graphene is thus obtained to be *κ* = 562.07 ± 77.54 W m^−1^ K^−1^ and the obtained interface conductance is *g* = 9.14 ± 1.74 MW m^−2^ K^−1^. These values are in very good agreement with the literature for mechanically exfoliated multi-layer graphene.^3,8^

We now consider the case of CVD graphene samples for unwrinkled and wrinkled sites. Fig. 4(c) and (d) show the FEA simulation results of temperature rise with energy density utilizing 100× objective and the ratio (*T*_50×_/*T*_100×_) respectively for unwrinkled sites of CVD graphene. The thermal conductivity of unwrinkled regions was obtained as ≈165 W m^−1^ K^−1^ with an interface conductance of ≈26 MW m^−2^ K^−1^. Unlike the case of mechanically exfoliated graphene, the FEA simulations only yielded approximate values since the curve intersections in the *κ*–*g* plane were only obtained within error bars of the experimentally measured temperature rises, rather than for the experimental average value of temperature rises. We concur that a slight laser power dependence of the *κ* and/or *g* in the unwrinkled sites of CVD graphene may be responsible for the same. Fig. 4(e) shows FEA results for the temperature rise of the wrinkled location at 100× objective for different combinations of *κ* and *g*. The corresponding simulated ratio of temperature rise (*T*_50×_/*T*_100×_) is plotted in Fig. 4(f). The experimental temperature rise and temperature rise ratio (*T*_50×_/*T*_100×_) are obtained from Fig. 2(c) and (f) respectively. The temperature rise with 50× objective is shown in Fig. S7 (see the ESI).† By following the previously described extraction procedure, thermal conductivity and total interface conductance values for wrinkled few-layer graphene are obtained as *κ* = 223.4 ± 20.0 W m^−1^ K^−1^ and *g* = 1.83 ± 1.60 MW m^−2^ K^−1^. To summarize the observations, the thermal conductivity measured for unwrinkled sites of CVD graphene was found to be 70% lower than the estimated value for mechanically exfoliated multi-layer graphene. However, the wrinkle sites of CVD graphene showed a locally enhanced thermal conductivity, which was higher by ≈35% when compared to regions devoid of wrinkles. At the same time, the wrinkle sites were associated with very low interface thermal conductance when compared to the other samples.

We now discuss the physical origin of the thermal conductivity in our CVD graphene samples, with particular reference to the wrinkle-mediated enhancement. A significantly lower value of *κ* for CVD graphene as compared to that for exfoliated graphene can arise from defects in the former. CVD graphene is typically associated with atomic-scale defects and grain boundaries introduced during the growth process and these defects serve as phonon scatterers. Usually, a large decrease in the thermal conductivity of graphitic systems is associated with a decrease in grain size.^37^ In all our samples, however, the defect peak is small, *I*_D_/*I*_G_ < 0.09. Attributing the defects to be edge-type, the grain-size, *L*_a_, is obtained using the expression, *L*_a_ = 2.4 × 10^−10^*λ* (*I*_D_/*I*_G_)^−1^.^38^ For unwrinkled regions of CVD graphene, *I*_D_/*I*_G_ (unwrinkled) = (3.36 ± 0.14) × 10^−2^, hence the calculated value of grain size, *L*_a_ = (572.02 ± 24.99) nm. Similarly, for wrinkled regions *I*_D_/*I*_G_ (wrinkled) = (8.47 ± 0.30) × 10^−2^, hence the calculated value of grain size, *L*_a_ = (226.96 ± 7.94) nm. This grain size is large enough and introduces relatively smaller defect density by the grain boundaries so that it may not serve as the heat transport limiting factor. This brings up the question, is there a factor different from defects that is responsible for the lower thermal conductivity of unwrinkled regions in CVD graphene? The observed decrease in thermal conductivity for unwrinkled regions of CVD graphene samples can have important contributions from stacking faults present in the sample, which can lead to an increase in the phonon scattering rate.^16,39^ Considering the larger grain size in our samples, enhanced phonon scattering can arise if the relative orientation of the few-layer stacks remains intact over relatively larger distances allowing for supercell level periodicity. With stacking faults the size of the unit cell increases leading to a consequent decrease in the size of the first Brillouin zone in the Fourier space. As a consequence, several folded phonon-branches appear in the reduced Brillouin zone. This can cause an increase in the phonon–phonon scattering and hence causes a decrease in thermal conductivity in the CVD samples, which comprise vertical assembly of few-layer crystallites with rotational stacking faults.^16,39^

Finally, we discuss the interesting observation of wrinkle-mediated local enhancement of thermal conductivity in our CVD graphene samples. In the literature, wrinkles have been predicted to suppress rather than enhance the thermal conductivity of single-layer graphene.^18,19,21^ This effect arises from phonon localization and is directly related to the curvature of the wrinkle. Thus, depending on the local curvature and on the stacking of layers, wrinkles can influence thermal transport in quite different ways. It is thus important to consider the height aspect ratio of the wrinkle along the shorter direction. This aspect ratio for wrinkles in our samples was found to be ≈11.36. In comparison, the theoretical literature which predicts phonon localization effects at the wrinkle sites of single-layer graphene had considered a smaller wrinkle aspect ratio of ≈2.80.^19^ Since our sample has a comparatively lower wrinkling level (<10%) and lower associated stress, any reduction in thermal conductivity from the pristine value may be correspondingly smaller based on the curvature of the wrinkle. The significant decrease in the total interface conductance value compared to that of mechanically exfoliated multi-layer graphene is in agreement with the fact that the wrinkle location is in poor contact with the substrate/base layer. Depending on the bending rigidity and film thickness, 2D materials tend to release the residual stresses by bending in the out-of-plane direction. These residual stresses in the film are caused by various factors including thermal expansion coefficient mismatch between the film and substrate, transfer procedures, edge stresses *etc.* In this way, partial and local detachment of the film from the substrate can take place. Again wrinkles by themselves are greatly stressed/compressed along the texture direction where as they are stretched along the wrinkling direction. This curvature in the film at the wrinkle sites or the local departure of the flexible sheets of graphene from equilibrium configuration can lead to local modulation of thermal properties. In our CVD graphene samples, the height of the wrinkle is itself comparable to the film thickness and the width is also in the order of few hundred nano-meters. Hence a decrease in the total interface conductance of the wrinkled location is in reconciliation with the fact that the contact between the substrate and the film at the wrinkle location has substantially reduced. This leads to a consequent decrease of heat transfer between the film and substrate. Both the model data and experimental results are in good agreement with this fact. Concomitant with the partial suspension of the film at the wrinkle sites are local and partial lifting of the stacking faults^16^ In other words, we suggest that the vertical crystallites get spatially decoupled when the film is locally delaminated. This partially restores the thermal conductivity whose original decrease had an important contribution from rotational stacking faults between the crystallites. Indeed, *κ* for suspended 5-layer graphene was estimated to be high, ∼1500 W m^−1^ K^−1^ by Ghosh *et al.*^8^ We thus suggest that the plausible explanation of wrinkle-mediated enhanced thermal conductivity lies in the local delamination and decoupling of the vertical crystallite assemblies.

## 4 Conclusion

Altered morphologies like wrinkling and stacking faults are ubiquitous in layered 2D materials. They can be deliberately induced or sometimes they result from synthesis and processing conditions. Understanding of their effects on thermal transport is useful for practical devices. In the literature, curvature or wrinkling is suggested to suppress thermal conductivity in single-layer graphene by localizing phonons. However, a remarkable wrinkle-mediated enhancement of thermal conductivity is observed in our CVD graphene films, where the associated wrinkle curvatures were relatively smaller. The CVD graphene films were characterized by vertical assemblies of few-layer graphene crystallites with stacking disorder, leading to a decrease in thermal conductivity. However, thermal conductivity at the wrinkle sites showed local enhancement, and we attribute this to wrinkle-mediated uncoupling or detachment of the mis-stacked layers together with partial suspension of the film. The total interface conductance at wrinkle locations was also decreased compared to that of their unwrinkled and mechanically exfoliated counterparts which clearly reiterates the departure of the film from local mechanical equilibrium and reduction of the interface contact. We demonstrate that the interplay of rotational stacking faults and wrinkling can substantially tune thermal transport in a graphene-based system. Our results are useful towards understanding heat transport in thermal coatings based on layered 2D materials, where departures from ideal morphologies can be commonly observed.

## Conflicts of interest

The authors declare no competing financial interests.

## Supplementary Material

NA-003-D0NA00944J-s001
