# Supplementary material for: Thermal transport across wrinkles in few-layer graphene stacks
Source: Nanoscale Adv. 2021 Jan 13;3(6):1708–16. doi: 10.1039/d0na00944j (PMC9417215; doi:10.1039/d0na00944j)
Supplement: NA-003-D0NA00944J-s001 [file NA-003-D0NA00944J-s001.pdf]

Electronic Supplementary Material (ESI) for Nanoscale Advances.
This journal is © The Royal Society of Chemistry 2021

Thermal transport across wrinkles in few-layer graphene stacks

A. Mohapatra,[1,2] S. Das,[3] K. Majumdar,[3] M. S. Ramachandra Rao,[2,†] and Manu Jaiswal[1,*]

[1]Department of Physics, Indian Institute of Technology Madras, Chennai 600036, India

[2]Department of Physics, Indian Institute of Technology Madras, Chennai 600036, India

[3]Department of Electrical Communication Engineering, Indian Institute of Science, Bangalore 560012, India

Email: *manu.jaiswal@iitm.ac.in; †msrrao@iitm.ac.in

I. AFM thickness profiles of mechanically exfoliated and wrinkled regions of CVD grown graphene

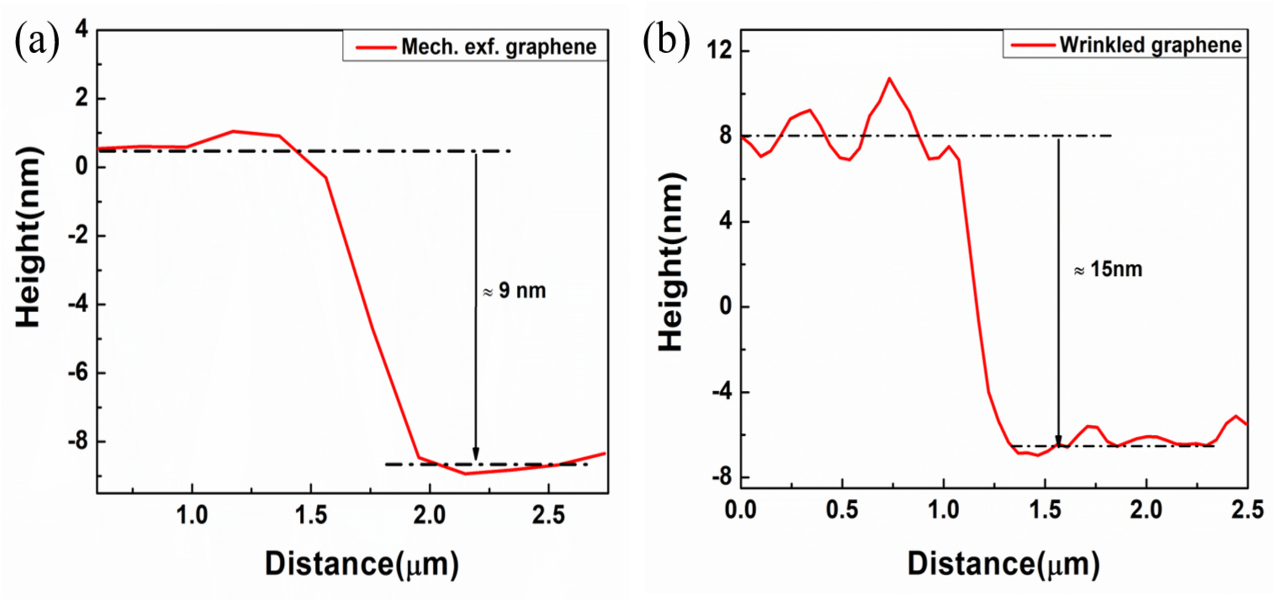

Figure S1: (a) AFM thickness profile of mechanically exfoliated multi-layer graphene measured along the red-line as indicated in Fig. 1(a) of the main text, (b). Thickness profile of wrinkled graphene locations in CVD grown samples, measured along the red-line as indicated in Fig. 1(b) of the main text

Fig. S1(a) shows the AFM thickness profile image of mechanically exfoliated multi-layer graphene along the red-line as shown in Fig. 1(a) (see main text). Estimated thickness from the thickness profile is obtained to be ≈ 9 nm. Exfoliated graphene at such thickness shows absence of wrinkles, which can be attributed to the high bending rigidity associated with the film which varies as cube of the thickness.[1] Fig. S1(b) shows the thickness profile of wrinkled regions of CVD graphene measured along the red-line as indicated in Fig. 1(b) (see main text). Thickness of wrinkled locations is estimated to be ≈ 15 nm.

II. Raman spectroscopy study with different incident laser wavelengths

Fig. S2(a) shows the Raman 2D peak of wrinkled locations obtained using two different laser wavelengths. Raman spectra marked with different color are indicative of the laser wavelength used in the measurement. Spectrum marked with green color shows the 2D peak obtained with laser wavelength 532 nm (2.33 eV). Similarly spectrum indicated in red color is obtained with laser wavelength of 633 nm (1.95 eV). A comparison of the line-

shape and deconvolution of 2D mode obtained with either wavelengths is consistent with the signatures of 5-layer graphene.[2,3,4,5]. From these observations it is inferred that wrinkled regions of CVD graphene consist of stacks of 5-layer graphene crystallites arranged vertically with stacking disorder, as also studied rigorously in the literature.[2,3,4,5] Fig. S2(b) shows the Raman 2D peak of unwrinkled regions of CVD graphene obtained using two different incident laser wavelengths. The lineshape of the peaks and the deconvolution are consistent with the signatures of 4-layer graphene. This shows that unwrinkled regions of CVD graphene consists of stacks of 4-layer graphene crystallites arranged vertically with stacking disorder.

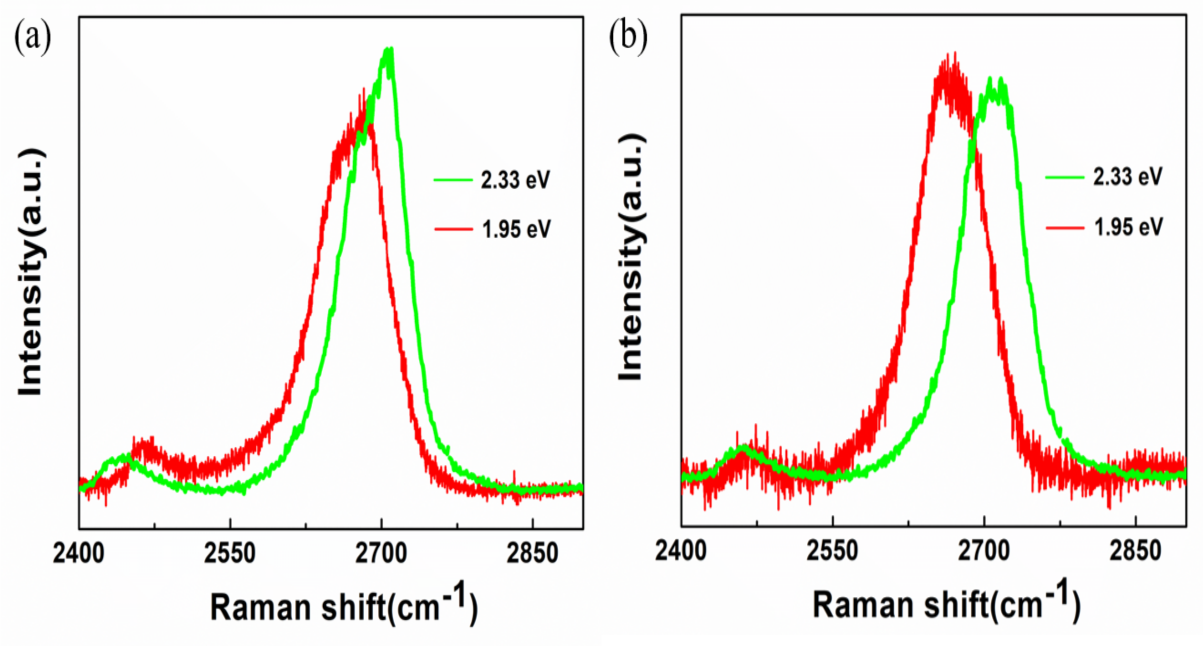

Figure S2: Raman 2D peak with two different incident laser wavelengths at 2.33 eV (green) and 1.95 eV (red) for: (a) wrinkled region of CVD grown sample, (b) unwrinkled region.

III. AFM topography and Raman spectroscopy study of unwrinkled regions of CVD grown graphene

Fig. S3(a) shows the AFM topography image of unwrinkled locations in our CVD grown samples. Thickness profile of unwrinkled locations is shown in Fig. S3(b) with an estimated thickness $\approx$ 13.5 nm. Fig. S3(c) shows the 2D peak of Raman spectrum taken at unwrinkled locations. The line shape and deconvolution as shown in the Fig. S3(c) is consistent with the signatures of 4-layer graphene.[2,3,4,5] Hence unwrinkled few-layer graphene locations consists of stacks of 4-layer graphene crystallites which are vertically arranged with stacking disorder.

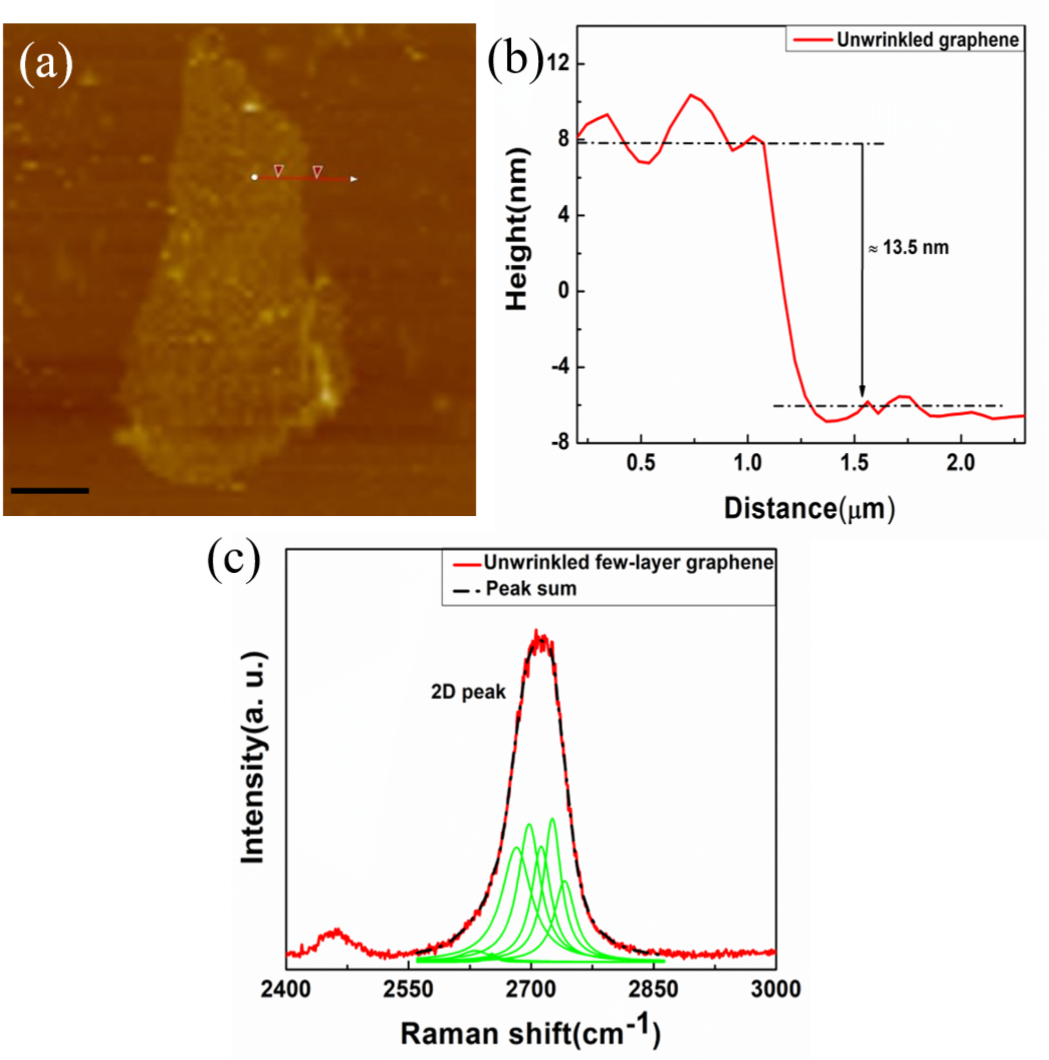

Figure S3: (a) AFM topography image of unwrinkled region of CVD graphene (scale bar = 2 μm), (b) AFM height profile image of unwrinkled region taken along the redline as indicated in 'part-a' (c) 2D band of unwrinkled region with its deconvolution.

IV. Raman maps of wrinkled and unwrinkled few-layer graphene

All the Raman maps were collected using 0.2s acquisition time with 1s of retracing time over a grid of 80×80 lines within an area of 25×15 μm^2. Fig. S4(a), (b) show the 2D-peak area-integrated Raman map and standard deviation Raman map of CVD graphene containing wrinkles, respectively. Similarly, Fig. S4(c), (d) show the 2D-peak area-integrated Raman map and standard deviation Raman map of unwrinkled CVD graphene, respectively. There is scarcely any variation in the Raman intensity and standard deviation of the 2D peak throughout the surface of the films. This indicates that the layer number variation in our samples is small, as discussed in the main text.

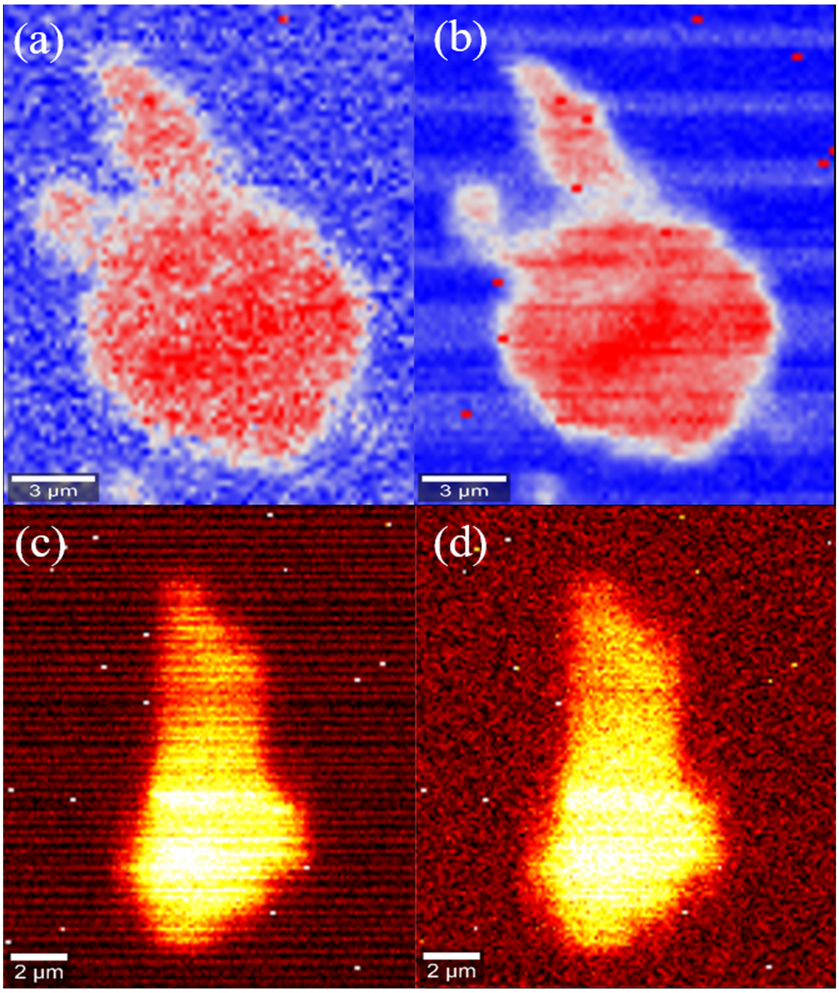

Figure S4: (a) 2D-peak area-integrated Raman map of CVD graphene containing randomly oriented uniaxial wrinkles and (b) 2D-peak standard deviation Raman map of the same region. (c) 2D-peak area-integrated Raman map of unwrinkled CVD graphene and (d) 2D-peak standard deviation Raman map of the same.

V. TEM-SAED analysis of stacking disorder in few-layer graphene samples grown using CVD technique

Graphene samples synthesized using CVD technique using camphor as solid precursor, always showed Raman signatures corresponding to few-layer graphene despite the large thickness revealed in AFM. This was attributed to the presence of rotational misalignment between the vertically stacked few-layer graphene crystallites. For further confirmation of the same, transmission electron microscopy measurements were performed using Tecnai G20 transmission electron microscope.

TEM sample preparation: Graphene grown on Ni substrate was wet transferred onto TEM grids (Cu grid - 200 mesh) as described below. Graphene on Ni substrate was spin-coated with PMMA solution at 2500 RPM for 40 sec. followed by another cycle at 2000 RPM for 40 sec. for better adhesion between the polymer layer and graphene. The sample was

subsequently heated at 70°C for 30 minutes. Nickel etchant solution was prepared using HCl + H_2O_2 (2M+1M) solution in DI water. After the etching process was completed in $\sim$ 2 hours, the sample was initially dipped into DI water followed by acetone for PMMA removal for $\sim$ 12 hours. It is then thoroughly rinsed with DI water for 5-6 times. Finally, the samples were scooped on to the TEM grids.

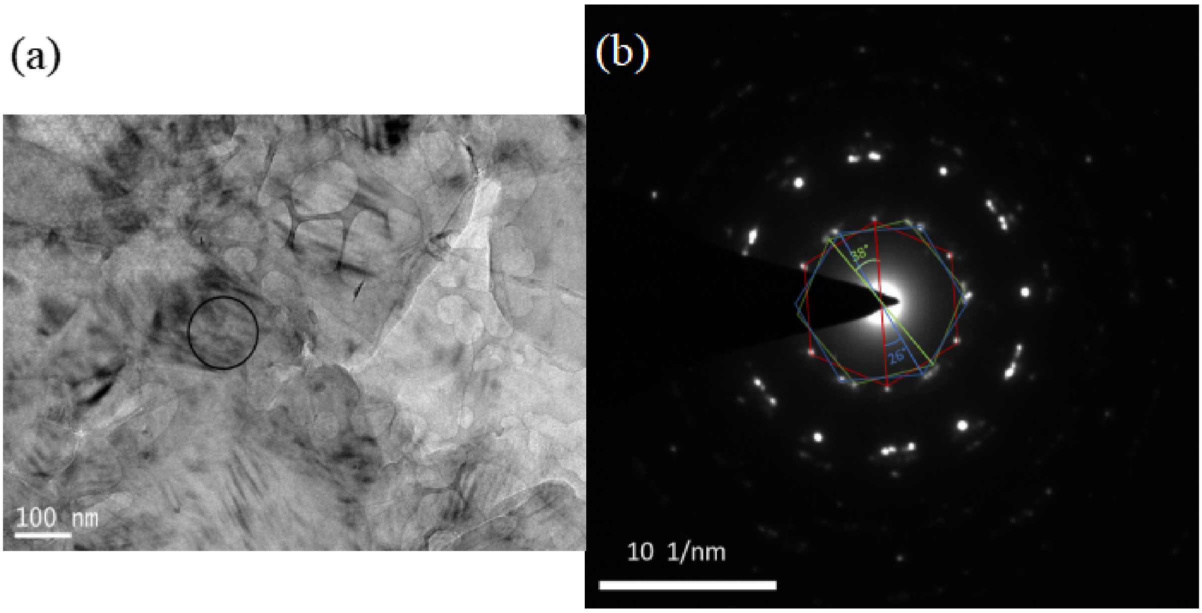

Figure S5: (a). TEM micrograph of the CVD synthesized graphene sample, (b). Representative SAED pattern, obtained from the region marked with black circle in part '(a)'.

Figure S5(a) shows the TEM micrograph of the transferred sample in bright field configuration with an incident beam energy of 200 keV. The region marked with black circle shows the position from where the representative diffraction signal is collected. Selected Area Electron Diffraction (SAED) has been used extensively in literature to reveal the rotational misorientation between stacked graphene layers.[6,7,8,9] Figure S5(b) shows the corresponding SAED image acquired from the region marked in part-(a). Hexagonal diffraction pattern can be seen corresponding to the hexagonal symmetry associated with the graphene lattice. Three sets of hexagonal reflection spots which are rotated with respect to each other are observed, as marked with different colored hexagons in Fig. S5(b). This illustrates the relative rotation about the c-axis between vertical crystallite stacks, with rotation angles of 38° and 26° observed at this location and variable other rotational angles are observed from other locations. This further confirms the observations made from Raman spectroscopy. To note, TEM-SAED analysis has been used in literature to show rotational misorientation in graphene systems including in incommensurate graphene foam grown by CVD,[7] misoriented CVD bilayers,[8] and misoriented few-layer graphene grown by magnetic field modulated DC carbon arc.[6] Finally, we note that few-layer graphene samples synthesized using camphor as solid precursor, always showed the presence of rotational stacking mis-orientation, as studied

by Kalita et. al.[10] Our graphene synthesis technique is quite identical to that of Kalita et al., as discussed in the main text.

VI. Details of instrument calibration procedure

The G peak shift was measured with different vertical positions (Z), and the shift was found to be maximum at Z = 3 μm position for 50x and Z = 2 μm for 100x objectives. This instrumental offset error was accounted by defining the position associated with maximal G-shift as $Z_0 = 0$ μm.[11] We examined objective calibration carefully and no lateral change in the laser spot position with change in the objective was observed. Thus, the same spatial region is in focus for both the objectives.

VII. Details of FEA simulation

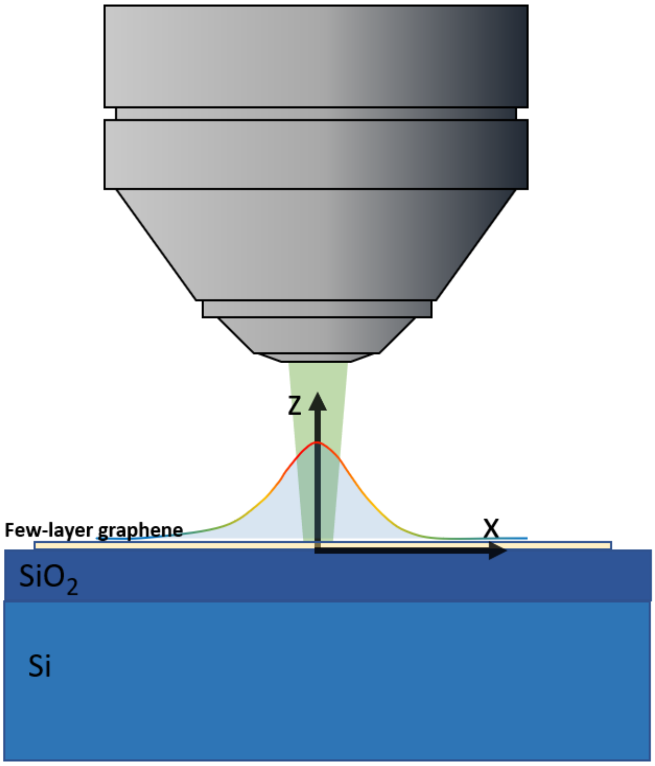

Figure S6: Schematic diagram showing Raman optothermal measurement of supported few-layer graphene and its temperature rise with the incident excitation laser.

Fig. S6 shows the schematic diagram of the system used in our finite element simulation. A laser beam heats the sample and the local temperature rise is optically measured from the shifts in phonon mode frequencies. Here we used the incident laser with excitation wavelength 532 nm. Energy distribution of the laser beam is Gaussian in both the directions

x and y. The laser spot is modelled by a Gaussian beam with the intensity given as:

$$I = \frac{I_0}{\pi {r_0}^2} exp(-\frac{x^2 + y^2}{{r_0}^2}) \tag{1}$$

Here r_0 is the beam spot size and I_0 is the total incident intensity. Graphene samples were transferred onto Si/SiO_2 substrates and the model shows a schematic representation of the thin-film configuration. For convenience, the interface between few-layer graphene and SiO_2 is chosen as Z = 0 plane. During the measurement, the fundamental peak of silicon did not show any shift, which is indicative of the fact that temperature rise at Si/SiO_2 interface is negligible. This condition serves as one of the boundary conditions in our model for the substrate (see the main text). κ_{sub} is chosen as 1.37 W/mK. We used an incident power of 1 mW for our calculations and obtained the temperature rise of the film ΔT for a set of given combination of κ and g utilizing two different power densities obtained using 50× and 100× objectives. As the incident power density associated with 100× objective is higher than 50×, the temperature rise obtained with 100× is obtained to be higher. The average temperature rise T_m is calculated for each objective. The ratio of the temperature rise is given as, $T_{50\times}/T_{100\times} = \chi_{50\times}/\chi_{100\times}$. Hence, the temperature rise ratio was used in conjunction with magnitude of temperature rise to determine κ and g uniquely. The experimental temperature rise of the film was obtained from the slopes of temperature variation and power variation measurements following the equation: $T_m = P_0 \times \left(\chi_P/\chi_T\right)$ The experimental temperature rise and temperature rise ratio in combination with FEA simulation results was utilized for the calculation of κ and g uniquely.

VIII. Finite element simulation results showing temperature rise of wrinkled and unwrinkled regions of CVD graphene

Fig. S7(a) shows the temperature rise of mechanically exfoliated multi-layer graphene with 50× objective. The temperature rise for unwrinkled region of CVD grown samples with 50× objective is shown in Fig. S7(b). Similarly, for wrinkled region of CVD graphene, the temperature rise with 50× objective is shown in Fig. S7(c). Fig. S7(d) shows the temperature rise of the unwrinkled locations with 100× objective at higher values of total interface conductance. As it can be seen from Fig. S7(d), the temperature rise is constant and parallel to x axis for higher values of g. It indicates to the fact that thermal conductivity associated with experimental temperature rise in Fig. S7(d) has a well defined lower limit.

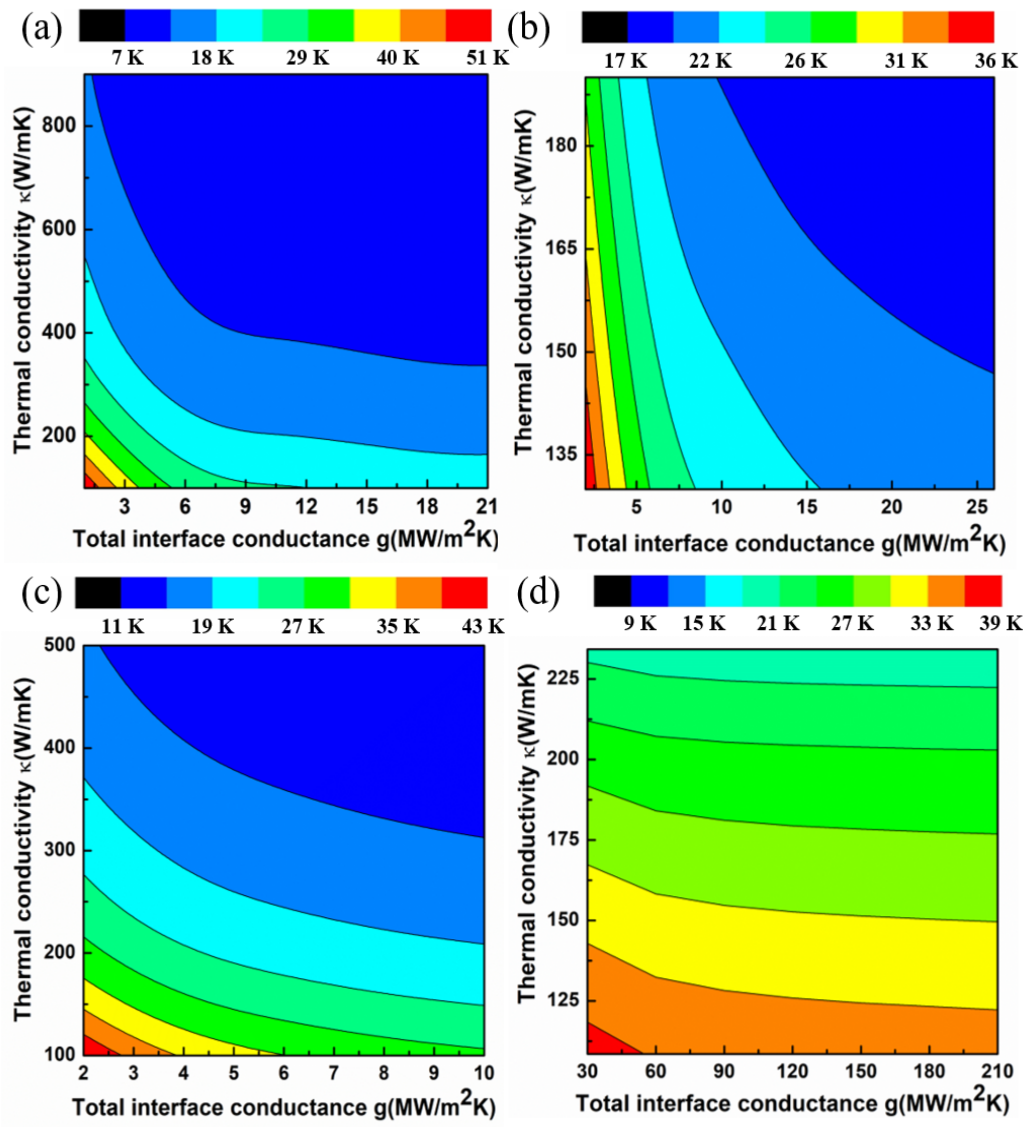

Figure S7: FEA simulation results showing temperature rise with 50× objective for (a) mechanically exfoliated multi-layer graphene, (b) unwrinkled region of CVD graphene, (c) wrinkled region of CVD graphene. (d) Temperature rise in unwrinkled regions plotted for higher values of total interface conductance

References

[1] Landau, L.; Lifshitz, L.; Theory of elasticity; **1970**, Oxford, New York.

[2] Nguyen, T.; Lee, J.; Yoon, D. and Cheong H.; Excitation energy dependent Raman signatures of ABA and ABC-stacked few-layer graphene; *Sci Rep*, **2014**, *4*, 4630

[3] Wu, J. B.; Lin, M. L.; Cong, X.; Liu, H. N. and Tan, P. H.; Raman spectroscopy of graphene-based materials and its applications in related devices; *Chem. Soc. Rev.*, **2018**, *47*, 1822-1873

[4] Poncharal, P.; Ayari, A.; Michel, T. and Sauvajol, J.-L.; Effect of rotational stacking faults on the Raman spectra of folded graphene; *Phys. Rev. B*, **2009**, *79*, 195417

[5] Ferrari, A. C.; Meyer, J. C.; Scardaci, V.; Casiraghi, C.; Lazzeri, M.; Mauri, F.; Piscanec, S.; Jiang, D.; Novoselov, K. S.; Roth, S. and Geim, A. K.; Raman spectrum of graphene and graphene layers; *Phys. Rev. Lett.*, **2006**, *97*, 187401

[6] Karmakar, S.; Nawale, A. B.; Lalla, N. P.; Sathe, V. G.; Kolekar, S. K.; Mathe, V. L.; Das, A. K. and Bhoraskar, S. V.; Gas phase condensation of few-layer graphene with rotational stacking faults in an electric-arc; *Carbon*, **2013**, *55*, 209 - 220

[7] Paronyan, T.; Thapa, A.; Sherehiy, A.; Jasinski J. B. and Jangam J. S. D.; Incommensurate graphene foam as a high capacity lithium intercalation anode; *Sci Rep*, **2017**, *7*, 39944

[8] Yamazaki, K.; Maehara, Y. and Gohara, K.; Characterization of TEM moiré patterns originating from two monolayer graphenes grown on the front and back sides of a copper substrate by CVD method; *Journal of the Physical Society of Japan*, **2018**, *87*, 061011

[9] David, L.; Bhandavat, R.; Kulkarni, G.; Pahwa, S.; Zhong, Z. Singh, G.; Synthesis of graphene films by rapid heating and quenching at ambient pressures and their electrochemical characterization; *ACS Applied Materials & Interfaces*, **2013**, *5*, 546-552

[10] Kalita, G.; Wakita, K. and Umeno, M.; Structural analysis and direct imaging of rotational stacking faults in few-Layer graphene synthesized from solid botanical precursor; *Japanese Journal of Applied Physics*; **2011**, *50*, 070106

[11] Judek, J.; Gertych, A. P.; Swiniarski, M.; Lapinska, A.; Duzynska, A. and Zdrojek, M.; High accuracy determination of the thermal properties of supported 2D materials; *Sci. Rep*, **2015**, *5*, 12422
